# Supplementary material for: The causal relationship between the human gut microbiota and pyogenic arthritis: a Mendelian randomization study
Source: Front Cell Infect Microbiol. 2024 Nov 26;14:1452480. doi: 10.3389/fcimb.2024.1452480 (PMC11629706; doi:10.3389/fcimb.2024.1452480)
Supplement: Supplementary file 1 [file DataSheet1.zip › mendelian test/ebi-a-GCST90027719.csv_leave_one_out.pdf]

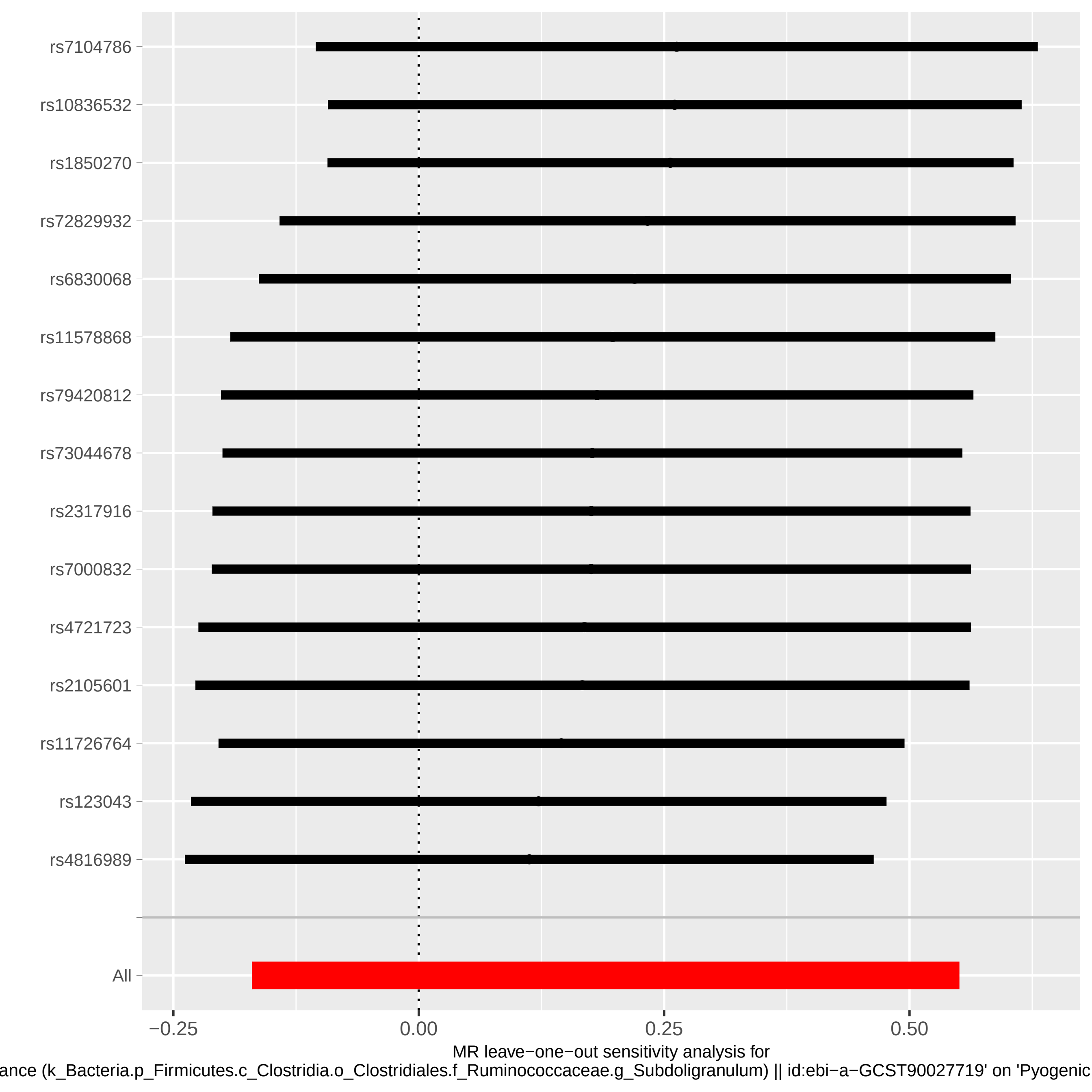

rs7104786
rs10836532
rs1850270
rs72829932
rs6830068
rs11578868
rs79420812
rs73044678
rs2317916
rs7000832
rs4721723
rs2105601
rs11726764
rs123043
rs4816989
All
−0.25
0.00
0.25
0.50
MR leave−one−out sensitivity analysis for
ance (k_Bacteria.p_Firmicutes.c_Clostridia.o_Clostridiales.f_Ruminococcaceae.g_Subdoligranulum) || id:ebi−a−GCST90027719' on 'Pyogenic
